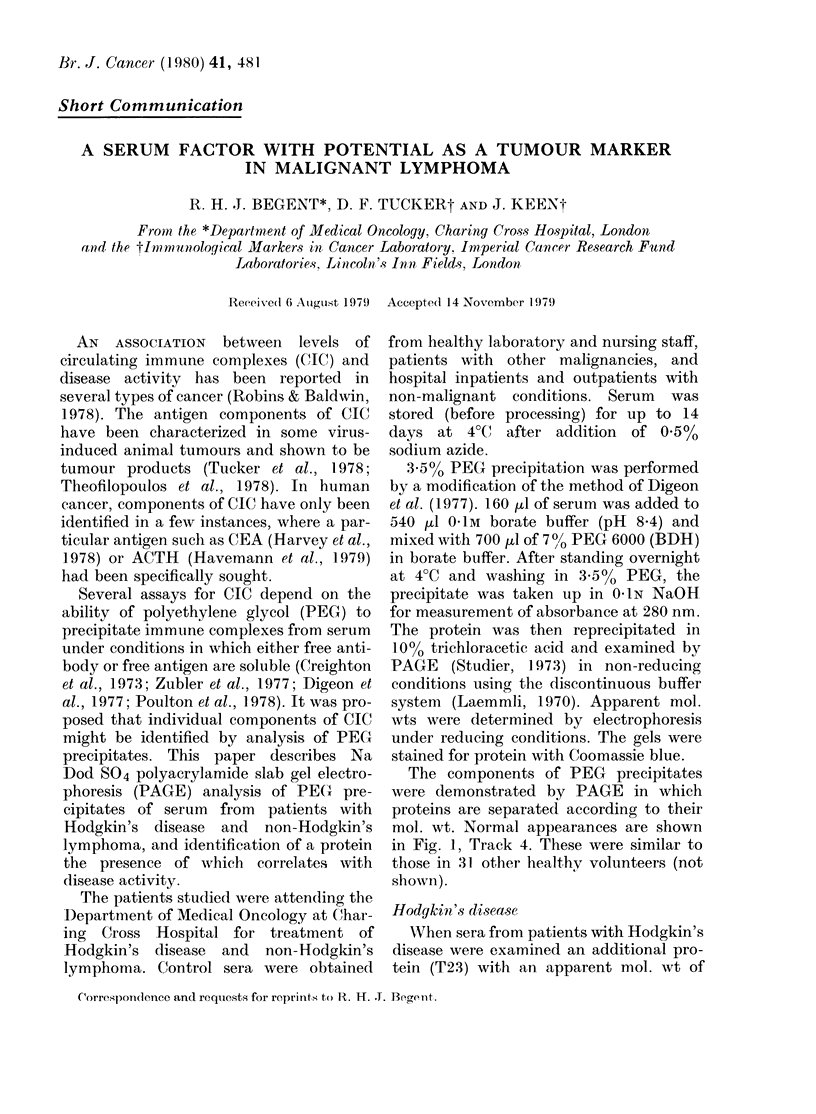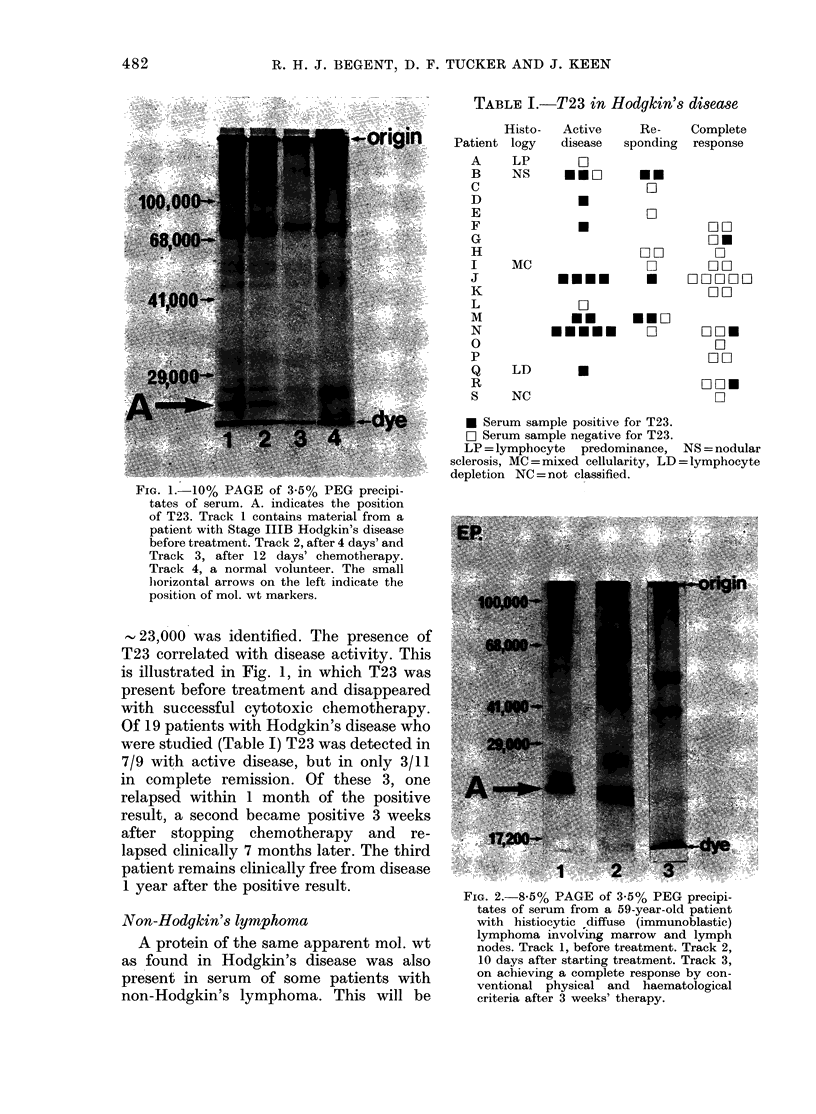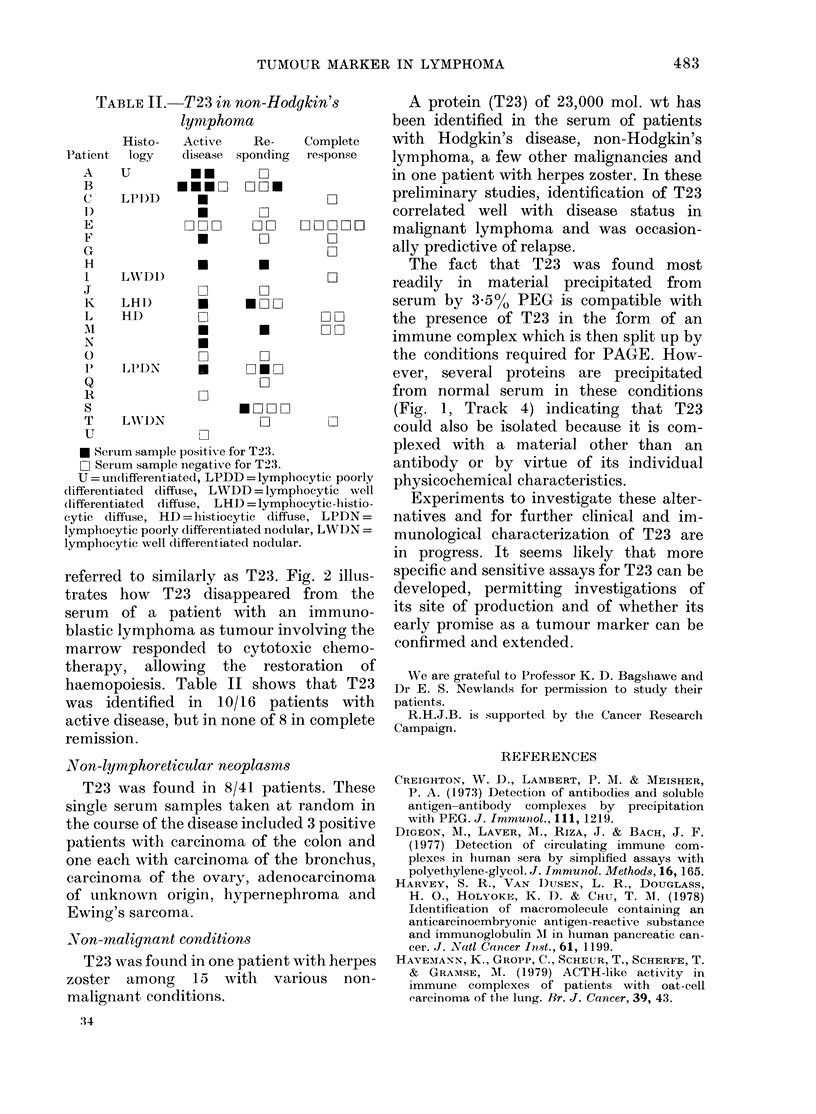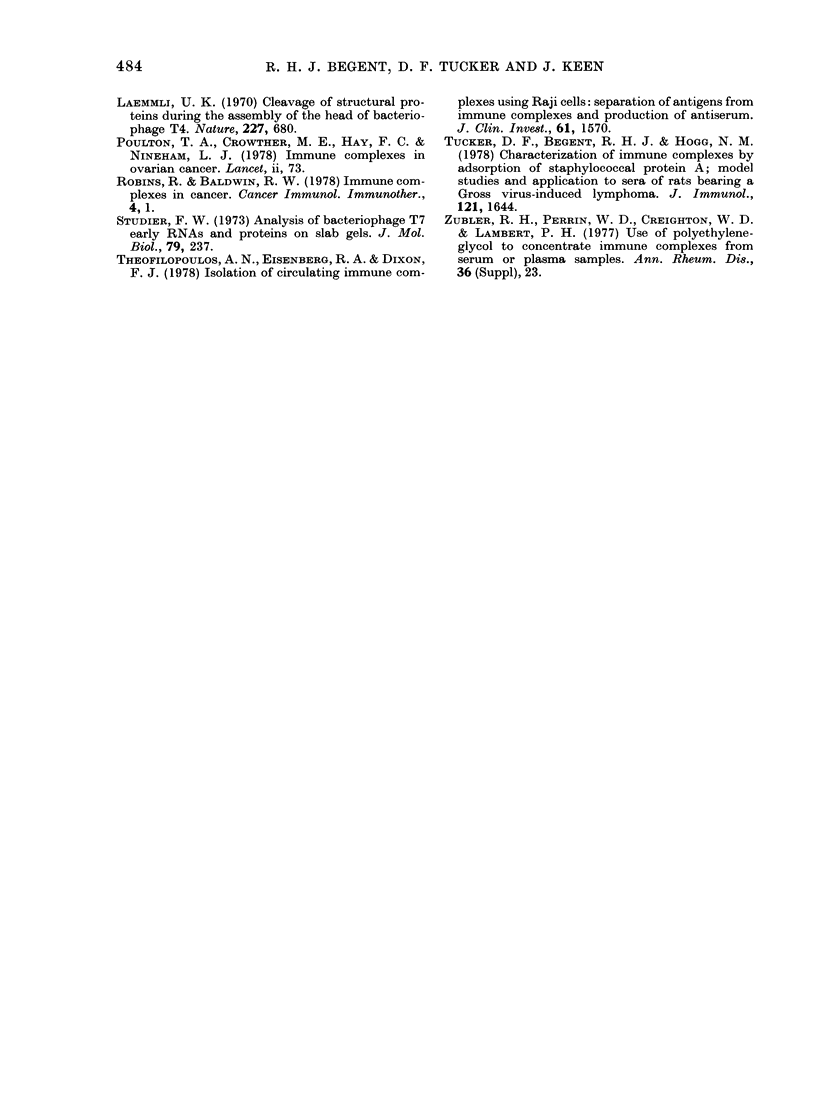# A serum factor with potential as a tumour marker in malignant lymphoma.

**DOI:** 10.1038/bjc.1980.74

**Published:** 1980-03

**Authors:** R. H. Begent, D. F. Tucker, J. Keen

## Abstract

**Images:**


*Br. J. Cancer* (1980) **41**, 481

***Short Communication***

### A SERUM FACTOR WITH POTENTIAL AS A TUMOUR MARKER IN MALIGNANT LYMPHOMA

R. H. J. BEGENT*, D. F. TUCKER† AND J. KEEN†

*From the *Department of Medical Oncology, Charing Cross Hospital, London and the †Immunological Markers in Cancer Laboratory, Imperial Cancer Research Fund Laboratories, Lincoln's Inn Fields, London*

Received 6 August 1979 Accepted 14 November 1979

AN ASSOCIATION between levels of circulating immune complexes (CIC) and disease activity has been reported in several types of cancer (Robins & Baldwin, 1978). The antigen components of CIC have been characterized in some virus-induced animal tumours and shown to be tumour products (Tucker *et al.*, 1978; Theofilopoulos *et al.*, 1978). In human cancer, components of CIC have only been identified in a few instances, where a particular antigen such as CEA (Harvey *et al.*, 1978) or ACTH (Havemann *et al.*, 1979) had been specifically sought.

Several assays for CIC depend on the ability of polyethylene glycol (PEG) to precipitate immune complexes from serum under conditions in which either free antibody or free antigen are soluble (Creighton *et al.*, 1973; Zubler *et al.*, 1977; Digeon *et al.*, 1977; Poulton *et al.*, 1978). It was proposed that individual components of CIC might be identified by analysis of PEG precipitates. This paper describes Na Dod $SO_4$ polyacrylamide slab gel electrophoresis (PAGE) analysis of PEG precipitates of serum from patients with Hodgkin's disease and non-Hodgkin's lymphoma, and identification of a protein the presence of which correlates with disease activity.

The patients studied were attending the Department of Medical Oncology at Charing Cross Hospital for treatment of Hodgkin's disease and non-Hodgkin's lymphoma. Control sera were obtained from healthy laboratory and nursing staff, patients with other malignancies, and hospital inpatients and outpatients with non-malignant conditions. Serum was stored (before processing) for up to 14 days at 4°C after addition of 0·5% sodium azide.

3·5% PEG precipitation was performed by a modification of the method of Digeon *et al.* (1977). 160 μl of serum was added to 540 μl 0·1M borate buffer (pH 8·4) and mixed with 700 μl of 7% PEG 6000 (BDH) in borate buffer. After standing overnight at 4°C and washing in 3·5% PEG, the precipitate was taken up in 0·1N NaOH for measurement of absorbance at 280 nm. The protein was then reprecipitated in 10% trichloracetic acid and examined by PAGE (Studier, 1973) in non-reducing conditions using the discontinuous buffer system (Laemmli, 1970). Apparent mol. wts were determined by electrophoresis under reducing conditions. The gels were stained for protein with Coomassie blue.

The components of PEG precipitates were demonstrated by PAGE in which proteins are separated according to their mol. wt. Normal appearances are shown in Fig. 1, Track 4. These were similar to those in 31 other healthy volunteers (not shown).

### *Hodgkin's disease*

When sera from patients with Hodgkin's disease were examined an additional protein (T23) with an apparent mol. wt of

Correspondence and requests for reprints to R. H. J. Begent.

FIG. 1.—10% PAGE of 3·5% PEG precipitates of serum. A. indicates the position of T23. Track 1 contains material from a patient with Stage IIIB Hodgkin's disease before treatment. Track 2, after 4 days' and Track 3, after 12 days' chemotherapy. Track 4, a normal volunteer. The small horizontal arrows on the left indicate the position of mol. wt markers.

~23,000 was identified. The presence of T23 correlated with disease activity. This is illustrated in Fig. 1, in which T23 was present before treatment and disappeared with successful cytotoxic chemotherapy. Of 19 patients with Hodgkin's disease who were studied (Table I) T23 was detected in 7/9 with active disease, but in only 3/11 in complete remission. Of these 3, one relapsed within 1 month of the positive result, a second became positive 3 weeks after stopping chemotherapy and relapsed clinically 7 months later. The third patient remains clinically free from disease 1 year after the positive result.

*Non-Hodgkin's lymphoma*

A protein of the same apparent mol. wt as found in Hodgkin's disease was also present in serum of some patients with non-Hodgkin's lymphoma. This will be

TABLE I.—*T23 in Hodgkin's disease*

| Patient | Histology | Active disease | Responding | Complete response |
|---|---|---|---|---|
| A | LP | □ | | |
| B | NS | ■■□ | ■■ | |
| C | | | □ | |
| D | | ■ | | |
| E | | | □ | |
| F | | ■ | | □□ |
| G | | | | □■ |
| H | | | □□ | □ |
| I | MC | | □ | □□ |
| J | | ■■■■ | ■ | □□□□□ |
| K | | | | □□ |
| L | | □ | | |
| M | | ■■ | ■■□ | |
| N | | ■■■■■ | □ | □□■ |
| O | | | | □ |
| P | | | | □□ |
| Q | LD | ■ | | |
| R | | | | □□■ |
| S | NC | | | □ |

■ Serum sample positive for T23.
□ Serum sample negative for T23.
LP = lymphocyte predominance, NS = nodular sclerosis, MC = mixed cellularity, LD = lymphocyte depletion NC = not classified.

FIG. 2.—8·5% PAGE of 3·5% PEG precipitates of serum from a 59-year-old patient with histiocytic diffuse (immunoblastic) lymphoma involving marrow and lymph nodes. Track 1, before treatment. Track 2, 10 days after starting treatment. Track 3, on achieving a complete response by conventional physical and haematological criteria after 3 weeks' therapy.

TABLE II.—*T23 in non-Hodgkin's lymphoma*

| Patient | Histology | Active disease | Responding | Complete response |
|---|---|---|---|---|
| A | U | ■■ | □ | |
| B | | ■■■□ | □□■ | |
| C | LPDD | ■ | | □ |
| D | | ■ | □ | |
| E | | □□□ | □□ | □□□□□ |
| F | | ■ | □ | □ |
| G | | | | □ |
| H | | ■ | ■ | |
| I | LWDD | | | □ |
| J | | □ | □ | |
| K | LHD | ■ | ■□□ | |
| L | HD | □ | | □□ |
| M | | ■ | ■ | □□ |
| N | | ■ | | |
| O | | □ | □ | |
| P | LPDN | ■ | □■□ | |
| Q | | | □ | |
| R | | □ | | |
| S | | | ■□□□ | |
| T | LWDN | | □ | □ |
| U | | □ | | |

■ Serum sample positive for T23.
□ Serum sample negative for T23.
U = undifferentiated, LPDD = lymphocytic poorly differentiated diffuse, LWDD = lymphocytic well differentiated diffuse, LHD = lymphocytic-histiocytic diffuse, HD = histiocytic diffuse, LPDN = lymphocytic poorly differentiated nodular, LWDN = lymphocytic well differentiated nodular.

referred to similarly as T23. Fig. 2 illustrates how T23 disappeared from the serum of a patient with an immunoblastic lymphoma as tumour involving the marrow responded to cytotoxic chemotherapy, allowing the restoration of haemopoiesis. Table II shows that T23 was identified in 10/16 patients with active disease, but in none of 8 in complete remission.

*Non-lymphoreticular neoplasms*

T23 was found in 8/41 patients. These single serum samples taken at random in the course of the disease included 3 positive patients with carcinoma of the colon and one each with carcinoma of the bronchus, carcinoma of the ovary, adenocarcinoma of unknown origin, hypernephroma and Ewing's sarcoma.

*Non-malignant conditions*

T23 was found in one patient with herpes zoster among 15 with various non-malignant conditions.

A protein (T23) of 23,000 mol. wt has been identified in the serum of patients with Hodgkin's disease, non-Hodgkin's lymphoma, a few other malignancies and in one patient with herpes zoster. In these preliminary studies, identification of T23 correlated well with disease status in malignant lymphoma and was occasionally predictive of relapse.

The fact that T23 was found most readily in material precipitated from serum by 3·5% PEG is compatible with the presence of T23 in the form of an immune complex which is then split up by the conditions required for PAGE. However, several proteins are precipitated from normal serum in these conditions (Fig. 1, Track 4) indicating that T23 could also be isolated because it is complexed with a material other than an antibody or by virtue of its individual physicochemical characteristics.

Experiments to investigate these alternatives and for further clinical and immunological characterization of T23 are in progress. It seems likely that more specific and sensitive assays for T23 can be developed, permitting investigations of its site of production and of whether its early promise as a tumour marker can be confirmed and extended.

We are grateful to Professor K. D. Bagshawe and Dr E. S. Newlands for permission to study their patients.

R.H.J.B. is supported by the Cancer Research Campaign.

REFERENCES

CREIGHTON, W. D., LAMBERT, P. M. & MEISHER, P. A. (1973) Detection of antibodies and soluble antigen–antibody complexes by precipitation with PEG. *J. Immunol.*, **111**, 1219.

DIGEON, M., LAVER, M., RIZA, J. & BACH, J. F. (1977) Detection of circulating immune complexes in human sera by simplified assays with polyethylene-glycol. *J. Immunol. Methods*, **16**, 165.

HARVEY, S. R., VAN DUSEN, L. R., DOUGLASS, H. O., HOLYOKE, K. D. & CHU, T. M. (1978) Identification of macromolecule containing an anticarcinoembryonic antigen-reactive substance and immunoglobulin M in human pancreatic cancer. *J. Natl Cancer Inst.*, **61**, 1199.

HAVEMANN, K., GROPP, C., SCHEUR, T., SCHERFE, T. & GRAMSE, M. (1979) ACTH-like activity in immune complexes of patients with oat-cell carcinoma of the lung. *Br. J. Cancer*, **39**, 43.

LAEMMLI, U. K. (1970) Cleavage of structural proteins during the assembly of the head of bacteriophage T4. *Nature*, **227**, 680.

POULTON, T. A., CROWTHER, M. E., HAY, F. C. & NINEHAM, L. J. (1978) Immune complexes in ovarian cancer. *Lancet*, ii, 73.

ROBINS, R. & BALDWIN, R. W. (1978) Immune complexes in cancer. *Cancer Immunol. Immunother.*, **4**, 1.

STUDIER, F. W. (1973) Analysis of bacteriophage T7 early RNAs and proteins on slab gels. *J. Mol. Biol.*, **79**, 237.

THEOFILOPOULOS, A. N., EISENBERG, R. A. & DIXON, F. J. (1978) Isolation of circulating immune complexes using Raji cells: separation of antigens from immune complexes and production of antiserum. *J. Clin. Invest.*, **61**, 1570.

TUCKER, D. F., BEGENT, R. H. J. & HOGG, N. M. (1978) Characterization of immune complexes by adsorption of staphylococcal protein A; model studies and application to sera of rats bearing a Gross virus-induced lymphoma. *J. Immunol.*, **121**, 1644.

ZUBLER, R. H., PERRIN, W. D., CREIGHTON, W. D. & LAMBERT, P. H. (1977) Use of polyethyleneglycol to concentrate immune complexes from serum or plasma samples. *Ann. Rheum. Dis.*, **36** (Suppl), 23.